# Bilateral anterior thalamic symmetrical infarction: a case study

**DOI:** 10.1186/s12883-023-03226-2

**Published:** 2023-05-06

**Authors:** Tong Wu, He Li, Qian Zhang, Wei Tang, Gehui Jia, Lei Li, Yong Zhang, Jiawei Wang

**Affiliations:** 1 Affiliated Hospital of Shandong University of Traditional Chinese Medicine, 250014 Jinan, China; 2 Shandong University of Traditional Chinese Medicine, 250355 Jinan, China; 3 Xuan Wu Hospital of the Capital Medical University, 100053 Beijing, China; 4 Dalian University Affiliated Xinhua Hospital, 16021 Shanghai, China

**Keywords:** Bilateral thalamic symmetrical infarction, Bilateral thalamic lesions, Cognitive function decreased, Percheron artery

## Abstract

**Background:**

Bilateral anterior thalamic symmetrical infarction is very rarely observed in clinical practice and has rarely been reported in the literature. In this paper we introduce a patient with bilateral anterior thalamic symmetrical infarction and discuss his symptoms, treatment process, and follow-up visit results, as well as the potential pathological mechanisms of the disease.

**Case presentation:**

: A 71-year-old male had a sudden cognitive decline four days prior to medical consultation. The patient’s brain MRI showed symmetrical high signals in the anterior part of both sides of the thalamus. The patient’s head MRV and immunological tests were normal, and we considered that this patient had a rare case of bilateral anterior thalamic infarction. After 10 days of anti-platelet aggregation that lowered blood lipids and improved circulation, the patient’s symptoms significantly abated. Two years later, we found through telephone follow-up that the patient’s symptoms had not relapsed substantially and that he was able to perform self-care, having only continued to suffer a slight decline in short-term memory.

**Conclusion:**

For patients with bilateral prethalamic lesions who have only acute cognitive impairment, if the lesions conform to the blood supply area of both thalamic nodular arteries and DWI shows a high signal, the diagnosis of acute cerebral infarction should be considered, and the standard treatment plan for cerebral infarction should be given as soon as possible.

## Background

Bilateral anterior thalamic symmetrical infarction is very rarely seen clinically, and hence few reports on it have been published. This article reports the clinical manifestations, diagnosis, treatment process, and follow-up results for a patient with bilateral anterior thalamic infarction, with the aim of introducing both the source of the blood supply of the anterior thalamus and the process of differential diagnosis of bilateral anterior thalamic infarction to clinicians. We hope that such an introduction will increase the recognition of neurologists of this kind of thalamic infarction and improve its diagnosis and treatment efficiency.

## Case presentation

**Fig. 1:**
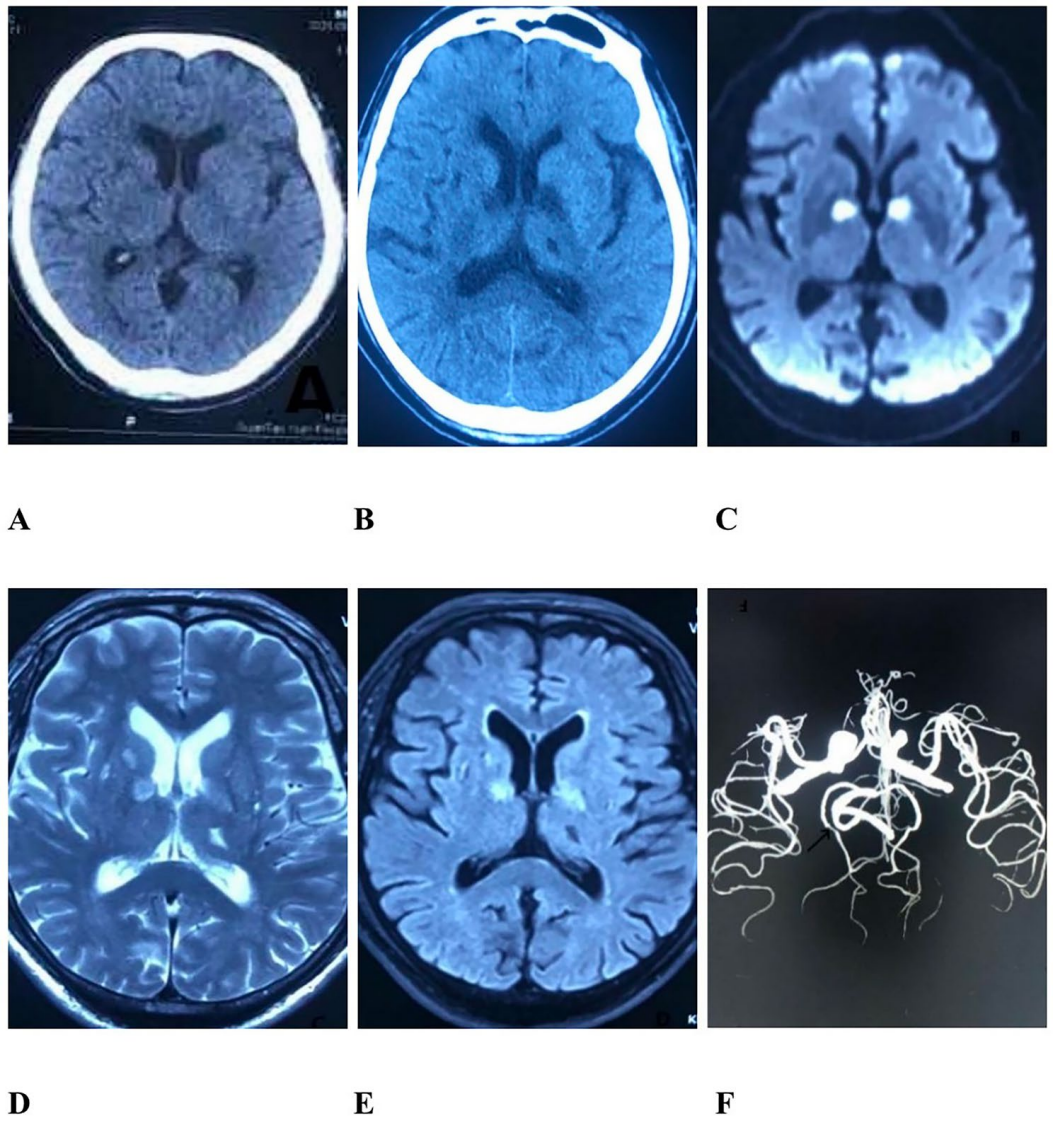
The patient’s imaging results (**A**) Head CT showed no obvious abnormalities (September 11, 2020). (**B**) Head CT showed bilateral low-density lesions in the anterior thalamus (September 12, 2020). (**C**-**E**) DWI, T2 weighted imaging, and FLAIR of the head showed symmetrical bilateral high signal lesions in the anterior part of the thalamus (September 12, 2020). (**F**) CTA of the head showed moderate to severe local stenosis of the P3 segment of the left posterior cerebral artery (the black arrow)

The patient in this case study is a 71-year-old male with a primary school education. On September 11, 2020, four days prior to medical consultation, his memory suddenly declined, and he couldn’t find his way home. He became unresponsive and his ability to comprehend the world around him decreased. In addition, the patient also appeared to suffer from a decline in language ability, an unchanging, indifferent expression, urinary and fecal incontinence, and an inability to recognize hunger and satiety. His motor function, sensory function, and swallowing function were not damaged. The patient was immediately sent to the hospital for emergency treatment by his family where he first underwent brain CT examination (Fig. 1-A) to rule out the possibility of cerebral hemorrhage. He then received thrombolytic therapy with urokinase (the specific dosage is unknown), but after this treatment his cognitive function did not improve. The patient then underwent brain CT and MRI examination one day later. CT showed that the patient had bilateral low density lesions in the anterior thalamus (Fig. 1-B). DWI showed symmetrical bilateral hyperintensities in the anterior thalamus (Fig. 1-C), as did T2 weighted imaging and FLAIR (Fig. 1-D, E).

The patient had a history of hypertension: his highest measured blood pressure was 150/90 mmHg, and his normal blood pressure was 130/70 mmHg. He did not take drugs regularly and had previously incurred a lateral thalamic infarction (lateral lesion on left thalamus Fig. 1B-E). The patient also had a history of smoking and drinking. Laboratory test results were as follows: D-dimer: 2.5ug/ml, plasma protein S: 72% (reference range: 77–143%), antithrombin III: 77% (reference range: 80–120%). There were no abnormalities found from routine blood, urine, stool tests, biochemical tests, hypersensitive C-reactive protein, erythrocyte sedimentation rate, thyroid function, tumor, anticardiolipin antibody, rheumatism, or immunity tests. However, carotid artery ultrasound showed bilateral carotid plaque formation, and CTA of head and neck showed moderate stenosis of the lower trunk of the right middle cerebral artery and moderate to severe local stenosis of the P3 segment of the left posterior cerebral artery (Fig. 1-F, black arrow). MRI venography (MRV) of the skull showed that the left transverse sinus and sigmoid sinus were both thin, which was considered to be the result of congenital development. No abnormalities were found during the fundus examination.

Physical examination of the patient’s nervous system after admission yielded the following results: clear mind, fluent speech, short-term memory decline, normal time orientation ability, normal place orientation ability, computational decline, normal object naming ability, normal language repetition ability, and normal distant memory function. Both nasolabial sulci were symmetrical, tongue extension occurred in the middle, and other cranial nerves were deemed normal. The Muscle strength of both limbs was Grade V, and muscle tension was normal, with bilateral tendon reflex (++) and bilateral Pap’s sign (-). The bilateral deep and shallow senses were normal, as was ataxia movement. Daily living ability was assessed as 75 points in accordance with the Barthel Index; the patient’s MMSE score was 6, his MoCA score was 2, and his clock drawing test score was 0. Finally, his Glasgow score (GCS) was 15; NIHSS score was 2 points (question 2); his Frog field drinking water test was Grade 1; and his Modified Rankin Scale (mRS) was Grade 3.

Interestingly, after ten days’ treatment with antiplatelet aggregation, which lowered his blood lipids and improved his circulation, the patient’s symptoms improved significantly. He could communicate simply, and his speech volume increased, although his reactions were slightly slow. At this point his NIHSS score was 1 point (question 1); his MRS was Level 2; and his Barthel Index was 85 points. The patient was subsequently discharged since his condition had improved. One month later, a telephone follow-up showed that his symptoms were significantly relieved, he was completely able to perform self-care. Only a slight short-term memory decline remained present. His mRS at this point was Level 1, and his Barthel Index was 90 points. Two years later, we found through telephone follow-up that the patient’s symptoms had not changed significantly and that he was still able to care for himself completely, with normal reaction time and only a slight decline in short-term memory. Here, his mRS score was Level 1, and his Barthel Index score was still 90 points.

## Discussion

The patient’s primary symptom was acute decline in short-term memory. Acute cognitive impairment is usually caused by injury to the temporal lobe, anterior frontal lobe, anteromedial thalamus, and corpus callosum and can be due to cerebrovascular diseases, encephalitis, infection, poisoning, and metabolic diseases [1]. The patient’s sudden illness, the existence of risk factors for cerebrovascular disease, and the fact that there was no evidence of metabolic disorder, infection, or poisoning all indicated that the possibility of cerebrovascular disease was high. The later DWI showed symmetrical bilateral lesions in the anterior part of the thalamus. This, combined with the fact that the patient evidently suffered from a speech disorder but was still able to read and retell, led us to conclude that his symptoms were consistent with thalamic dementia. The bilateral lesions in the anterior thalamus were thus deemed to be responsible for the patient’s cognitive impairment.

The anterior thalamic nucleus is located in the anterior part of the thalamus and is part of the Papez loop, which is related to memory, emotion, and intelligence and is supplied by thalamic tubercle artery [2]. The occlusion of the tuberous artery can therefore cause damage to the anterior thalamic nucleus, which can interrupt the connection between the anterior thalamic nucleus and the limbic system, leading to the decline of cognitive function. Cerebral infarction involving both sides of the thalamus is extremely rare. Previous relevant reports mostly refer to bilateral medial thalamus infarction caused by Percheron artery occlusion, but this only accounts for 0.1–0.2% of all cerebral infarction ^[[3, 4]]^. Bilateral anterior thalamic infarction is rarer and has not been reported in the literature thus far. Because few cases with only bilateral anterior thalamic infarction and no other infarctions have been reported before, such cases are very easy to be misdiagnosed as classic Percheron artery occlusion. However, Percheron artery occlusion, in most cases, results in bilateral medial thalamic infarction, and most of these cases have symptoms of consciousness disorder and vertical gaze paralysis [5]. The clinical manifestation of the patient in this case study is consistent with that of thalamic dementia [6], unconscious disorder, and abnormal gaze and is different from that of Percheron infarction. Furthermore, in terms of imaging results, the patient clearly has bilateral anterior thalamic infarction, which is consistent with the imaging results for the occlusion of the blood supply area of both thalamic nodular arteries [7].

There may be several reasons for the patient to have had bilateral anterior thalamus infarction. Some studies have reported that in a few patients, one or both thalamic nodular arteries are absent, and blood is instead supplied by the parathalamic median artery [8]. In addition, both parathalamic median areas having thalamic infarction is a type 4 Percheron infarction [9]. This patient only had lesions in the anterior part of the thalamus and did not have infarction in the parathalamic median area, so this patient did not conform to the typical pattern of type 4 Percheron infarction. This may be related to the partial reopening of the Percheron artery with urokinase after the early thrombolysis, which may have resulted in the infarction staying in the anterior part of the distal thalamus and not involving the medial side of the proximal thalamus. In addition, according to the patient’s clinical manifestations and imaging results, his tubercle artery may also have a common trunk, resulting in the bilateral thalamic infarction. Unfortunately, because the thalamic tubercle artery is relatively small, existing DSA, CTA, MRA and other angiographic techniques cannot show the existence and variation of this artery. At present, the diagnosis of which vessels are responsible for thalamic infarction is often inferred only through MRI results.

There are many diseases that can cause bilateral thalamic lesions, such as intracranial venous thrombosis, dural arteriovenous fistula, inflammatory demyelinating disease, tick borne encephalitis, epidemic encephalitis B, glioma, and Wernicke encephalopathy [10–14]. Since few cases with only bilateral anterior thalamic infarction and no other infarctions have been reported, and since this patient had no other typical neurological deficits, it may have been easy to misdiagnose the patient with a toxicity injury or metabolic disease when seeing his imaging results, and this would have delayed his receipt of needed treatment. The patient had no history of alcoholism, had a “moderate” nutritional status, no infection, and no abnormal blood inflammatory index, biochemical index, immune index, head MRV, or fundus examination. In combination with the patient’s clinical manifestations and imaging results, intracranial infection, immunity, tumor, metabolic brain disease, intracranial vein embolism, and other diseases were thus excluded.

Previous studies on thalamic perforator artery infarction have all claimed that intravenous thrombolysis is the best treatment [8, 15]. After intravenous thrombolysis with antiplatelet aggregation, lowering blood lipid and blood pressure, the patient did not use Allison, and his cognitive function recovered quickly, leaving only slight damage to his short-term memory. So far, the patient has been followed up with for 2 years, and there has been no aggravation of any neurological deficit symptoms. The early use of intravenous thrombolytic therapy was responsible for partially reopening his blood vessels, so his clinical symptoms abated quickly, resulting in less permanent nerve damage.

Research has shown that the prognosis of thalamic infarction without midbrain infarction is generally good [7], which is consistent with this case, where the neurological deficit of this simple thalamic infarction improved significantly after conventional treatment of cerebral infarction with antiplatelet and lipid-lowering drugs. In a study involving 23 patients with Percheron infarction, 65% of the patients had a good prognosis [8], which was also consistent with the obvious improvement in neurological function in this patient. Thus, we conclude that acute cognitive impairment after bilateral anterior thalamic infarction may have a better prognosis quicker recovery time after conventional cerebral infarction treatments. Such patients should therefore be identified as soon as possible to avoid excessive examination and medication.

## Conclusion

Based on the above cases, we believe that the possibility of suffering from acute cerebral infarction is very high in patients with bilateral anterior thalamic lesions who only have acute cognitive impairment if the lesions are on both thalamic tubercle arteries and DWI shows hyperintensities. Early active treatment strategies, especially intravenous thrombolytic therapy, may improve the prognosis of patients with such thalamic infarctions.

## Data Availability

The data used is available from the corresponding author upon reasonable request.

## Abbreviations

CT: Computed tomography
MRI: Magnetic resonance imaging
DWI: Diffusion Weighted Imaging
CTA: Computed Tomographic Angiography

## References

[1] Hermann P, Zerr I. Rapidly progressive dementias - aetiologies, diagnosis and management. Nat Rev Neurol. 2022;18(6):363–76. 10.1038/s41582-022-00659-0. 10.1038/s41582-022-00659-0 PMC9067549 35508635

[2] Lee S, Kim DY, Kim JS, Kaur G, Lippmann S. Visual hallucinations following a left-sided unilateral tuberothalamic artery infarction. Innov Clin Neurosci. 2011;8(5):31–4. PMC3115769 21686146

[3] Musa J, Rahman M, Guy A, Kola E, Guy A, Hyseni F, Cobo A, Saliaj K, Bushati F, Ahmetgjekaj I. Artery of Percheron infarction: A case report and literature review. Radiol Case Rep. 2021;16(6):1271–1275. 10.1016/j.radcr.2021.02.059. 10.1016/j.radcr.2021.02.059 PMC8027104 33854662

[4] Donohoe C, Nia NK, Carey P, Vemulapalli V. Artery of Percheron Infarction: a case report of bilateral thalamic stroke presenting with Acute Encephalopathy. Case Rep Neurol Med. 2022;302022:8385841. 10.1155/2022/8385841. 10.1155/2022/8385841 PMC8986439 35399910

[5] Kheiralla O, Alghamdi S, Aljondi R, Tajaldeen A, Bakheet A. Artery of Percheron Infarction: A Characteristic Pattern of Ischemia and Variable Clinical Presentation: A Literature Review. Curr Med Imaging. 2021;17(5):669–674. 10.2174/1573405616666201130095801. 10.2174/1573405616666201130095801 33256583

[6] Liu R, Zhao Y, Yin H, Shi Z, Chen X. Dural arteriovenous fistula presenting as thalamic dementia: a case description with rare imaging findings. Quant Imaging Med Surg. 2022;12(5):3000–6. 10.21037/qims-21-1054. 10.21037/qims-21-1054 PMC9014146 35502375

[7] Schmahmann JD. Vascular syndromes of the thalamus. Stroke. 2003;34(9):2264–78. 10.1161/01.STR.0000087786.38997.9E. 10.1161/01.STR.0000087786.38997.9E 12933968

[8] Zhang B, Wang X, Gang C, Wang J. Acute percheron infarction: a precision learning. BMC Neurol. 2022;22(1):207. 10.1186/s12883-022-02735-w. 10.1186/s12883-022-02735-w PMC9166501 35659267

[9] Satei AM, Rehman CA, Munshi S. Bilateral Thalamic Stroke Arising From an Occlusion of the Artery of Percheron: Barriers to Diagnosis, Management, and Recovery. Cureus. 2021;13(11):e19783. 10.7759/cureus.19783. 10.7759/cureus.19783 PMC8693548 34956778

[10] Kalita J, Sachan A, Dubey AK, Jain N, Kumar S. A clinico-radiological study of deep cerebral venous thrombosis. Neuroradiology. 2022;64(10):1951–60. 10.1007/s00234-022-02938-5. 10.1007/s00234-022-02938-5 35462575

[11] Clarke L, Arnett S, Lilley K, Liao J, Bhuta S, Broadley SA. Magnetic resonance imaging in neuromyelitis optica spectrum disorder. Clin Exp Immunol. 2021;206(3):251–65. 10.1111/cei.13630. 10.1111/cei.13630 PMC8561702 34080180

[12] Silveira L, Allison D, Delahmetovic E, Muse J, Penar P. Bilateral Thalamic Glioma: A Case Report. Cureus. 2021;13(11):e19570. 10.7759/cureus.19570. 10.7759/cureus.19570 PMC8671068 34926042

[13] Ibatullin RA, Magzhanov RV, Usmanov IF. Dvustoronnee porazhenie talamusa pri kleshchevom entsefalite [Thalamic lesion in tick-borne encephalitis]. Zh Nevrol Psikhiatr Im S S Korsakova. 2022;122(8):154–158. 10.17116/jnevro2022122081154. 10.17116/jnevro2022122081154 36036418

[14] Nalbantoglu M, Ozturk-Tan O, Bayazıt N, Tayfun F. Percheron artery infarction in the differential diagnosis of acute confusional state with normal initial brain MRI. Acta Neurol Belg. 2016;116(1):73–5. 10.1007/s13760-015-0505-1. 10.1007/s13760-015-0505-1 26133949

[15] Santos M, Rodrigues A, Albuquerque A, Santos F, Bandeira A, Magalhães M, Banza M (2021). Artery of Percheron ischaemic stroke: a Classic Presentation of a rare case. Eur J Case Rep Intern Med.

